# Nuku, a family of primate retrocopies derived from *KU70*

**DOI:** 10.1093/g3journal/jkab163

**Published:** 2021-05-24

**Authors:** Paul A Rowley, Aisha Ellahi, Kyudong Han, Jagdish Suresh Patel, James T Van Leuven, Sara L Sawyer

**Affiliations:** 1 Department of Biological Sciences, University of Idaho, Moscow, ID 83844, USA; 2 Department of Molecular Biosciences, University of Texas at Austin, Austin, TX 78751, USA; 3 Department of Microbiology, College of Science & Technology, Dankook University, Cheonan 31116, Republic of Korea; 4 Center for Bio- Medical Engineering Core Facility, Dankook University, Cheonan 31116, Republic of Korea; 5 Center for Modeling Complex Interactions, University of Idaho, Moscow, ID 83844, USA and; 6 Department of Molecular, Cellular, and Developmental Biology, University of Colorado Boulder, Boulder, CO 80302, USA

**Keywords:** retrocopy, Ku70, primates, NHEJ

## Abstract

The gene encoding the ubiquitous DNA repair protein, Ku70p, has undergone extensive copy number expansion during primate evolution. Gene duplications of *KU70* have the hallmark of long interspersed element-1 mediated retrotransposition with evidence of target-site duplications, the poly-A tails, and the absence of introns. Evolutionary analysis of this expanded family of *KU70*-derived “*NUKU*” retrocopies reveals that these genes are both ancient and also actively being created in extant primate species. *NUKU* retrocopies show evidence of functional divergence away from *KU70*, as evinced by their altered pattern of tissue expression and possible tissue-specific translation. Molecular modeling predicted that amino acid changes in Nuku2p at the interaction interface with Ku80p would prevent the assembly of the Ku heterodimer. The lack of Nuku2p-Ku80p interaction was confirmed by yeast two-hybrid assay, which contrasts the robust interaction of Ku70p-Ku80p. While several *NUKU* retrocopies appear to have been degraded by mutation, *NUKU2* shows evidence of positive natural selection, suggesting that this retrocopy is undergoing neofunctionalization. Although Nuku proteins do not appear to antagonize retrovirus transduction in cell culture, the observed expansion and rapid evolution of *NUKUs* could be being driven by alternative selective pressures related to infectious disease or an undefined role in primate physiology.

## Introduction

Protecting the integrity of a cell’s genetic material is important for both survival as well as for ensuring the faithful transmission of genes to daughter cells. Thus, DNA repair genes are conserved throughout the evolutionary history of prokaryotes and eukaryotes, with homologs present in every major organismal clade. A prime example is the *KU70* gene, involved in DNA double-strand break repair mediated by nonhomologous end-joining (NHEJ). Human Ku70p and Ku80p together form the Ku heterodimer, a well-established initiator of the NHEJ pathway for DNA double-strand break repair (Roth *et al.* 1985; Milne *et al.* 1996; Gu *et al.* 1997; Jin and Weaver 1997). In addition to its well-documented role in the NHEJ pathway, Ku70p is also involved in V(D)J recombination (Nussenzweig *et al.* 1996; Gao *et al.* 1998), telomere maintenance (Gravel *et al.* 1998; Hsu *et al.* 1999), Bax-mediated apoptosis (Subramanian *et al.* 2005), innate immune signaling (Zhang *et al.* 2011; Ferguson *et al.* 2012; Sui *et al.* 2017), and even cell–cell adhesion and extracellular matrix remodeling at the cell membrane (Monferran *et al.* 2004; Martinez *et al.* 2005; Chan *et al.* 2009). The *KU70* and *KU80* genes are present in eukaryotic and archaeal genomes, while in bacteria the role of the heterodimer is performed by a homodimer of the protein Ku (Aravind and Koonin 2001; Weller *et al.* 2002).

Gene duplication is an important mechanism by which new genes arise. After gene duplication, multiple possible fates await the new gene copy, depending on the selective forces at play: decay, purifying selection, subfunctionalization, or neofunctionalization (Long *et al.* 2003; Schlötterer 2015). Retrocopies (previously known as “processed pseudogenes”) are a type of gene duplication created when retrotransposons erroneously reverse transcribe a cellular mRNA and insert the cDNA copy of the gene back into the host genome (Kaessmann *et al.* 2009). As a result, retrocopies often lack introns (Long and Langley 1993; Schacherer *et al.* 2004; Benovoy and Drouin 2006). In addition, they can also be flanked by target-site duplications (TSDs), as is the case for mammalian LINE-1 mediated retrotransposition (Maestre *et al.* 1995; Esnault *et al.* 2000). Retrotransposition and the subsequent formation of retrocopies are cited as having had a singular effect on primate and human evolution, with a so-called “burst” in retrocopy formation during the last 63 million years have contributed to the emergence of many novel genes (Ohshima *et al.* 2003; Zhang *et al.* 2003). Approximately 3771–18,700 retrocopies of human genes exist in the human genome, with about 10% of these found to express mRNA transcripts (Harrison *et al.* 2005; Sisu *et al.* 2014; Casola and Betrán 2017).

The main *KU70*-related gene duplication that is known is the ancient duplication that gave rise to *KU70* and *KU80*, and thereby the eukaryotic Ku heterodimer. Here, we report the description of five *KU70* retrocopies in the human genome, which we have named *NUKU1–NUKU5*. Four of these retrocopies are present in all simian primate genomes, and therefore predate the split between Old World monkeys and New World monkeys over 30 million years ago (MYA). However, a newer retrocopy found on the human X chromosome, *NUKU5*, is specific to apes (human, gorilla, chimpanzee, and orangutan). *KU70* has spawned an unusual number of retrocopies, as it is the only one out of 66 genes linked to DNA double-strand break repair to have five retrocopies in the human genome. While the original open reading frames (ORFs) appear to be disrupted by mutations and indels, there is evidence for expression of *NUKU2*, *NUKU4*, and *NUKU5* and a spliced transcript that exists for *NUKU2*. *NUKU2* has also evolved under positive selection, and functional tests of *NUKU* genes and molecular modeling simulations reveal that it has functionally diverged from *KU70* in two ways. First, whereas *KU70* is expressed in all tissues, *NUKU2*, *NUKU4*, and *NUKU5* display a tissue-specific expression pattern and these transcripts are associated with ribosomes. Second, while Ku70p interacts with Ku80p, Nuku2p does not. Given the extensive functional characterizations of human *KU70* and *KU80* that have occurred over decades, it will now be of great interest to determine what potential role these additional *KU70*-like genes play in human biology.

## Materials and methods

### Identification and classification of retrocopies

The *KU70* coding sequence was used as a query in the UCSC genome browser against the human genome (http://genome.ucsc.edu/, March 2006 NCBI36/hg18 assembly, Last accessed 5.24.21). Six top hits of Blat scores were identified, the topmost of which matched with 100% sequence identity to the original *KU70* gene. The next five hits appeared as retrocopies upon closer inspection. *NUKU* orthologs from chimpanzee, orangutan, and rhesus macaque were also obtained using this method. For inspection of insertion sites in the marmoset genome, the calJac1 and calJac3 assemblies were used. All other insertion sites were interrogated using the current version of primate genomes found on the UCSC genome browser (https://genome.ucsc.edu). The phylogenetic trees of *KU70* and *NUKU* sequences were built with MEGA v.7 (maximum likelihood method) (Hall 2013) from DNA alignments built using MUSCLE (v3.8) and were manually curated to ensure accuracy.

The GO term “double-strand break repair” was queried in the GO database (GO term ID 0006302). Because not all genes have been fully annotated and assigned to appropriate GO categories (leading to exclusion of certain relevant genes from this list), we combined genes assigned to this GO category in either *Homo sapiens, Mus musculus*, and *Rat norvegicus.* This resulted in a list of 66 genes (Supplementary Table S1). cDNA coding sequences for all 66 hits were retrieved from NCBI. In the case of genes with multiple transcript variants or splicing variants, the longest transcript was used. To find retrocopies of each gene, cDNA sequences were used as queries in the UCSC human genome database (hg18). RetrogeneDB2 was also used in an attempt to identify *KU70* retrocopies in primates, but failed to identify *NUKU4* and was unable to identify any *KU70* retrocopies in primates and so was not used further (Rosikiewicz *et al.* 2017). Retrocopies were defined as hits in the human genome that met the following two criteria: (1) they lack introns (RepeatMasker was used to differentiate introns from transposable element insertions), and (2**)** they match the parent gene in a reciprocal best hit analysis of the human genome. Reciprocal best hit analysis was performed by taking each putative retrocopy and using the BLAST server at NCBI to query the human RefSeq mRNA database.

### Sequencing *KU70* and *NUKU* orthologs

*KU70* orthologs and *NUKU2* ORF orthologs were sequenced from mRNA-derived cDNA for *KU70* and from genomic DNA for *NUKU2* from 12 primates: gorilla (*Gorilla gorilla)*, agile gibbon (*Hylobates agilis*), colobus (*Colobus guereza*), crab-eating macaque (*Macaca fascicularis*), gibbon (*Pongidae Hylobates syndactylus*), leaf monkey (*Trachypithecus francoisi*), Borneo orangutan (*Pongo pygmaeus*), talapoin (*Miopithecus talapoin*), white-cheeked gibbon (*Nomascus leucogenys*), olive baboon (*Papio anubis*), black mangabey (*Ophocebus albigena*), and Wolf’s guenon (*Cercopithecus wolfi*) (Supplementary Table S2). Genes were PCR-amplified using the strategy described in Supplementary Table S3 and sequenced with primers shown in Supplementary Table S4. The full structure of the *NUKU2* transcript was determined with 5’ and 3’ RACE using the GeneRacer kit (Invitrogen), and testis total RNA (Ambion, catalog #7972). All nucleotide sequences are provided within Supplementary File S3.

### Evolutionary analysis of *KU70* retrocopies

Sequences of the human *KU70/NUKU* paralogs were collected from the UCSC genome browser and aligned using ClustalX. Sequences were analyzed under the free-ratio model implemented in the codeml program of PAML 3.14. In order to determine whether dN/dS > 1 on the *NUKU2* branch, we made a pairwise comparison between the ancestral (Anc) sequence (generated by codeml) and *NUKU2*. K-estimator (Comeron 1999) was used to run Monte Carlo simulations of neutral evolution of these sequences, creating a null distribution from which a *P*-value could be derived.

The branch-site test allows identification of positive selection that might be limited to a subset of codons along only a subset of the branches being analyzed (Zhang *et al.* 2005). To implement this test, multiple alignments were fitted to the branch-sites models Model A [positive selection model, codon values of dN/dS along background branches are fit into two site classes, one (ω_0_) between 0 and 1 and one (ω_1_) equal to 1, on the foreground branches a third site class is allowed (ω_2_) with dN/dS > 1], and Model A with fixed ω_2_ = 1 [null model, similar to Model A except the foreground ω_2_ value is fixed at 1). *NUKU2* branches (back to their last common ancestor] were defined as the “foreground” clade, with all other branches in the tree being defined as background branches. The likelihood of Model A is compared to the likelihood of the null model with a likelihood ratio test.

### *NUKU* expression in human tissues

Total RNA from human tissues was purchased from Clontech (catalog number 636643). Most of these samples represent pooled RNA from multiple individuals (between 2 and 63 individuals). First-strand cDNA was produced with the NEB Protoscript II kit (E6400S), using a dT_23_ primer that anneals indiscriminately to poly-A tails on mRNA molecules. First-strand reactions were carried out twice in parallel for each tissue, one with reverse transcriptase (RT), and one with water added instead of RT (indicated by ± RT on Figure 5). First-strand cDNA was then amplified with *KU70*- and *NUKU*-specific primers using Invitrogen PCR Supermix HiFi (cat 10790020). In order to increase specificity, two successive PCRs were performed. In the first round of PCR, 20 cycles were performed using primers specific to that gene, along with 2 µl of first-strand cDNA as template. In the second cycle, 0.5 µl of the first round PCR reaction was used as template, and one of the gene-specific primers was substituted with a nested primer (F2 or R2 in Figure 5B). In this round, amplification was performed for 40 cycles, and 2 µl of the final product was then run on a 2% agarose gel for separation. Primers used were: SS004 (Nuku F), SS011 (Nuku R1), SS009 (Nuku R2), SS030 (Ku70 F1), SS031 (Ku70 F2), and SS032 (Ku70 R) (Supplementary Table S4). The *KU70*-specific primers span an intron so that cDNA can be differentiated from the product that would be produced from genomic DNA. There are no introns in *NUKU*. Products were sequenced to confirm that they unambiguously represent *KU70* or *NUKU*.

### Molecular modeling of *NUKU2* using FoldX

To understand the effect of single missense variation on Ku70p stability (*i.e.*, folding) and its binding with Ku80p, we estimated both folding and binding ΔΔ*G* values (difference of free energies between wild-type and the mutant) using FoldX software (Schymkowitz *et al.* 2005). To run FoldX calculations, the X-ray crystal structure of the human Ku heterodimer was first downloaded from Protein Data Bank (PDB id: 1JEQ) (Walker *et al.* 2001). The file was modified to remove all but the two chains of Ku70p and Ku80p. Several residues were missing in both the chains of the protein complex. These missing residues were not modeled to complete the structure of the complex before running FoldX calculations for the following two reasons: (1) Missing residues were either at the terminal ends or in the disordered region hence they are difficult to build using the molecular modeling software and (2) the gaps in the input X-ray structure does not affect the performance of the FoldX software as it relies on rotamer libraries to model any mutation at a particular site and semi-empirical scoring function to estimate ΔΔ*G* values (Guerois *et al*. 2002). The clean starting structure of Ku70p-Ku80p complex was then used to create mutant models and subsequently estimate both binding ΔΔ*G* and folding ΔΔ*G* values. We started by performing 6 rounds of minimization of the protein complex using the RepairPDB command to obtain convergence of the potential energy. All 19 possible single amino acid mutations at each site on Ku70p (548 amino acid residues × 19 possible substitutions) were then generated using BuildModel. Finally, folding ΔΔ*G* values were estimated using Stability command on Ku70 and AnalyseComplex command was used to estimate the effect of each modeled mutation on Ku70p-Ku80p binding *i.e.*, binding ΔΔ*G* values.

### Yeast two-hybrid assay

We used the LexA-Gal4 yeast two-hybrid system, which employs the LexA DNA-binding domain (DBD) and the Gal4-activation domain (Gal4-AD) with the yeast strain EAY1098 (*His3, Leu2, Trp1*, and genotype). If the candidate proteins interact, the DNA-binding domain and activation domain (AD) will be in close proximity and will be able to drive the transcription of a *HIS3* reporter gene downstream of the LexA promoter. The Clontech pGADT7 plasmid, which creates an N-terminal fusion protein between a gene of interest and the Gal4 AD, was engineered to carry the full 1830 bp coding sequence of human *KU70*. Another pGADT7 vector was engineered to carry the full 654 bp *NUKU2* ORF. The full-length coding sequence of human *KU80* (2199 bp) was cloned into the LexA expression vector pBTM116, which creates an N-terminal fusion protein between the inserted gene and the LexA DNA binding domain. All cloning was done with TA-vectors and plasmids compatible with the Gateway system (Invitrogen). EAY1098 was transformed using the standard lithium-acetate PEG transformation protocol with the following plasmid pairs: pGADT7-Ku70 and pLexA-Ku80; pGADT7-Nuku and pLexA-Ku80; pGADT7 and pLexA-Ku80; and pGADT7 and pLexA. Transformants were selected on leucine and tryptophan drop-out media to select for and stimulate expression of plasmids. After 2 days growth at 30°C, saturated cultures at an OD_600_ of 2.7–2.8 were diluted and plated onto media lacking histidine in addition to leucine and tryptophan to stimulate *HIS3* gene reporter expression. Growth was observed 3 days post-plating.

### Western blots

Thirty micrograms of denatured protein lysate were loaded onto 10% Tris-HCl polyacrylamide gels and then transferred onto a nitrocellulose membrane. Membrane was blocked overnight in 5% milk-TBS + 1% Tween and incubated the next day with a primary antibody directed against the Gal4-AD (1:5000 dilution; Clontech, cat # 630402) or against human Ku70p (1:1000 dilution; GeneTex, cat # GTX101820). The secondary antibody for Gal4 probes was goat anti-mouse-HRP (1:1500; Fisher, cat #32430), and for Ku70p probes was goat anti-rabbit-HRP (1:1500 dilution; Fisher cat. #32460). Signal was detected using ECL plus reagents (VWR cat #95040-056). For analysis of two-hybrid constructs, total protein from yeast strains was prepared using the glass-bead disruption method. Fifty mL yeast cultures were grown to OD_600_ 0.5–0.7 and were pelleted. This pellet was suspended in disruption buffer: 20 mM Tris-HCl, pH 7.9, 10 mM MgCl_2_, 1 mM EDTA, 5% glycerol, 0.3 M (NH_3_)SO_4_, with 1 mM DTT, 1 mM PMSF, and Protease inhibitor cocktail (Roche). Acid-washed glass beads were added, and cells were vortexed for a total of 10 minutes.

### Western Blot analysis of Ku70 retrocopies

Human brain and testis tissue total protein lysates were purchased from ProSci Incorporated (catalog numbers 1303 and 1313, respectively). HEK293T cells were grown in standard DMEM with 10% fetal bovine serum in 75 cm^3^ tissue culture flasks. Total protein was prepared using the reagents and protocol described in the Qiagen Mammalian Protein preparation kit. Protein was quantified using Pierce Coomassie Bradford Assay reagent. About 30 µg of protein was separated using polyacrylamide gel electrophoresis on a Tris-HCl gel and transferred to a nitrocellulose membrane. Membranes were blotted with 1:1000 dilution of the Ku70p antibody raised in rabbit (GeneTex XRCC6 antibody, Cat.# GTX101820). Secondary antibody of Goat anti-rabbit conjugated to horseradish peroxidase at 1:1500 dilution was used (Cat. #32460 Thermo Scientific Pierce Goat anti-Rabbit IgG, Peroxidase Conjugated). Maltose binding protein/hemagglutinin-tagged Nuku proteins were detected using an anti-HA peroxidase-conjugated monoclonal rat antibody [3F10; 12013819001 (Roche)].

### Virus infection assays

Human HEK293T (4 × 10^5^) and HeLa (4  ×  10^4^) cells were seeded in 12-well dishes (DMEM growth medium with 10% fetal bovine serum) and grown at 37°C with 5% CO_2_ for 24 hours until reaching a confluency of ∼75%. Each well was transiently transfected with 800 ng of plasmid encoding either human *NUKU5*, *NUKU2* or rhesus macaque *NUKU2* in addition to a transfection control plasmid expressing RFP. After 24 hours incubation, each well was trypsinized and the HEK293T (2  ×  10^5^) and HeLa (4  ×  10^4^) cells used to seed three wells of a 24-well dish. After 24 hours of incubation at 37°C (5% CO_2_) monolayers with a confluency of ∼50% were infected with VSV-G pseudotyped HIV, FIV, or MLV containing a GFP reporter gene as described previously (Stabell *et al.* 2016). After 48 hours, cells were trypsinized and fixed with 1% paraformaldehyde by incubating for 1 hour at 4°C. GFP and RFP positive transduced cells were detected by flow cytometry using appropriate compensation controls to account for spectral overlap of fluorophores.

### Ribo-seq analysis

Raw sequencing data were downloaded from Wand *et al.* (2020). The reads were quality filtered and trimmed using fastp v0.20.0 using default parameters then mapped to *KU70* and retrocopies from the respective species using bwa mem v0.7.17 with default parameters. Alignments were processed using Samtools v1.10 (-F 4). Reads mapping to more than one reference sequence were further investigated and removed by parsing the “XA” tags from the alignment files using scanBam from the Rsamtools v2.4.0 package in R v4.03. Plotting was done in ggplot v3.3.3.

### Data availability

The data underlying this article are available in the article and in its online supplementary material available at figshare: https://doi.org/10.25387/g3.14267312.

## Results

### Five *KU70* retrocopies in the human genome

**Figure 1:**
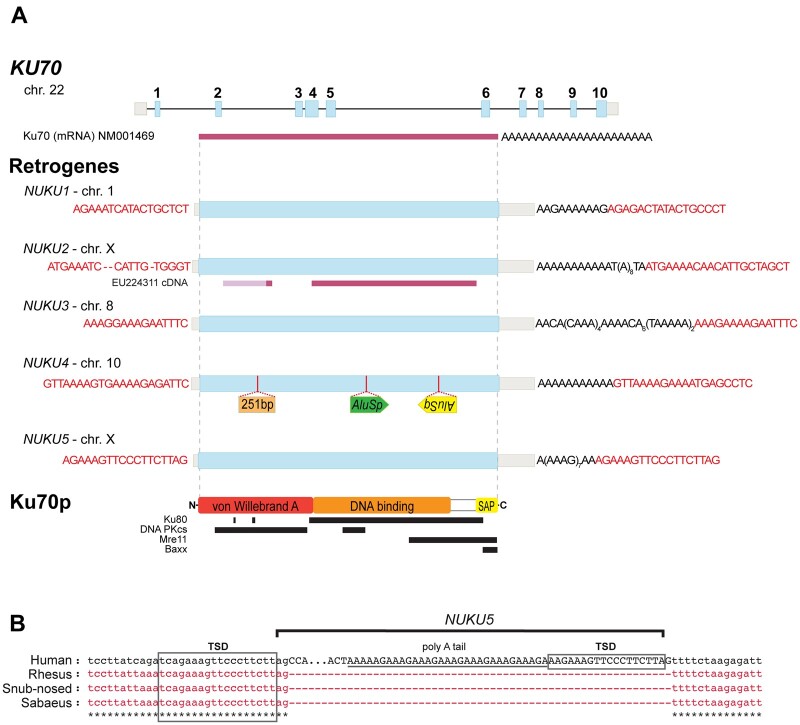
Identification of Five *KU70* Retrocopies in the Human Genome (A) A diagram of the *KU70* parent gene locus and the loci of its five retrocopies. Exons are shown in thick blue boxes and introns appear as black lines. 3’ and 5’ UTR structures are shown in light gray. TSD sequences are highlighted in red text. (B) Insertion of *NUKU5* in the human X chromosome compared to the syntenic locus of other Old World primates and evidence of LINE-1 mediated TSD.

Five ORFs with high similarity to *KU70* were identified on four different human chromosomes (Figure 1A). Unlike the human *KU70* gene locus, each of the five copies lack introns. TSDs characteristic of LINE-1 mediated insertion were identified flanking each of the retrocopies, as were 3’ poly-A tails that are relics of the mRNA from which these genes arose (Figure 1, A and B). All human retrocopies are between 89 and 97% identical to the parent *KU70* processed mRNA transcript and have been named *NUKU1–NUKU5*. Each of the five TSDs is unique, confirming that these copies represent five independent retrotransposition events, and did not arise from segmental duplication of an existing retrocopy-containing region. Thus, the human genome contains one *KU70* gene and five LINE-1 mediated *NUKU* retrocopies (Supplementary Table S5).

**Figure 2:**
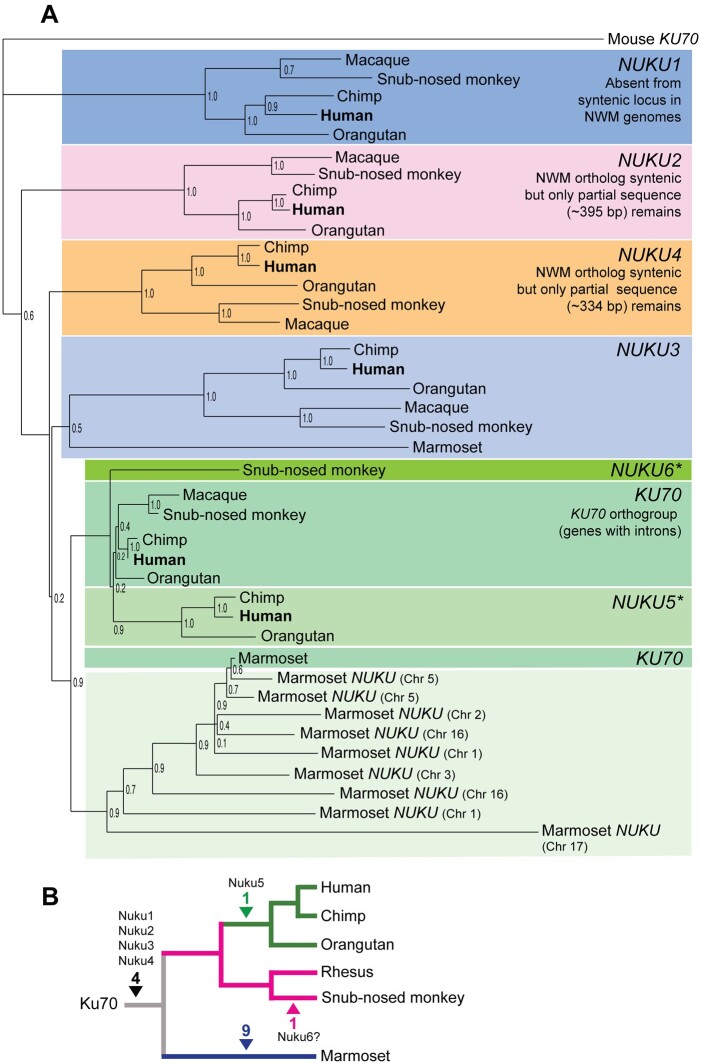
Phylogenetics and insertion sites of *KU70* derived retrocopies **(**A) Once the five *NUKU* retrocopies had been identified in the human genome, orthologous retrocopies were identified in other primate genome projects through inspection of the syntenic target sites. A tree of these sequences is shown. Unless indicated, none of the genes on the tree contain introns. Bootstrap values generated with the maximum likelihood method are shown. Marmoset *NUKU3* was verified to be orthologous to the other *NUKU3* sequences by target site analysis. *NUKU2* and *NUKU4* are apparent in the marmoset genome, but are almost completely deleted, and therefore they were not included in the alignment used to make the tree. We were unable to locate the syntenic region of *NUKU1* in the Marmoset genome, indicating that this region may have been deleted (Supplementary Figure S2). Marmoset-specific retrocopies were not named but are designated by the chromosome on which they are found. *target site of *NUKU5* and *NUKU6* insertion is empty in Old World monkeys. (B) Based on the phylogenetic analysis and target site inspection, *NUKU1–NUKU4* predate the split between Old World monkeys, New World monkeys, and hominoids. *NUKU5* is specific to the great ape genomes analyzed and *NUKU6* appears to be unique to the snub-nosed monkey. The time of acquisition of *NUKU6* is somewhat uncertain due to the low bootstrap support in the phylogenetic analysis. The marmoset genome has birthed 9 additional *KU70*-like retrocopies.

We then analyzed several primate genomes for the presence of *KU70* retrocopies (Supplementary Table S1). Phylogenetic analysis (Figure 2A and Supplementary Figure S1) and inspection of pre-insertion target sites (as in Figure 1B and Supplementary Figure S2) define the order in which these retrocopies arose, and places them at distinct positions in the tree of primate speciation (Figure 2B). These data show that four of the *KU70* retrocopies arose before the split between Old World and New World monkeys, over 30 MYA, consistent with a burst of retrocopy formation that has been reported in this time frame (Ohshima *et al.* 2003; Zhang *et al.* 2003; Marques *et al.* 2005). Remnants of *NUKU2* and *NUKU3* are present in the marmoset and squirrel monkey genomes, although they have experienced large subsequent deletions (Figure 2A and Supplementary Figure S2). We were unable to identify *NUKU1* in either the marmoset or squirrel monkey genomes (Supplementary Figure S2). Comparing the syntenic location of *NUKU1* in both marmoset and squirrel monkeys to the human genome reveals large indels that prevent the reconstruction of the evolutionary history of the locus in New World monkeys (Supplementary Figure S2). Because *NUKU1* is the most basally branching retrocopy, we predict that it also predates the last common ancestor of the species being analyzed. Interestingly, the genomes of both marmoset and squirrel monkeys have acquired many additional *KU70* retrocopies that are not found in any of the other primate genomes investigated, meaning that these arose after the last common ancestor of New World and Old World monkeys (30–40 MYA) (Figure 2 and Supplementary Figure S1). The human genome contains one new retrocopy, *NUKU5*, that is found in the genomes of chimpanzee and orangutan, but not in rhesus or marmoset. The pre-insertion site in the syntenic location in the rhesus macaque, snub-nosed monkey, and sabaeus monkey genomes are perfectly preserved (Figure 1B), confirming that this retrocopy post-dates the split between Old World monkeys and hominoids that occurred approximately 20 MYA. Analysis of the genome of golden snub-nosed monkey also revealed the birth of a new *KU70* retrocopy (*NUKU6*) with a TSD, remnants of a poly-A tail, which is absent from other Old World monkeys and humans (Supplementary Figure S3). This suggests that *NUKU6* is a unique retrocopy of *KU70.* Thus, *KU70* retrocopies have been consistently birthed over a period lasting more than 30 million years, with evidence of continued retrocopy birth in extant primate species (Supplementary Table S5).

None of the ORFs in any of the primate *NUKU* retrocopies have been conserved in their full-length form as compared to *KU70*, and at first glance they all appear to be retropseudogenes. *NUKU3*, located on chromosome 10, has acquired two *Alu* insertions (*Alu*Sp and *Alu*Sq elements) and a 251 bp insertion of non*KU70* related sequence in the middle of the coding region (Figure 1A). The ORF in *NUKU5* is approximately 75% the length of *KU70*, although *NUKU* ORFs are smaller, and the putative start codon of all of them is downstream of the *KU70* start codon.

### *KU70* has an unusually large number of retrocopies

**Figure 3:**
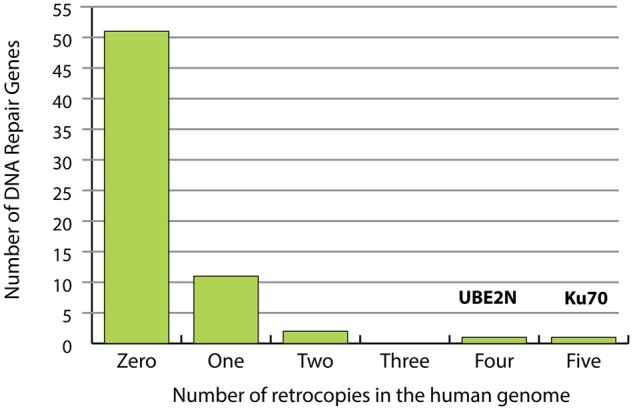
Prevalence of human retrocopies among double-strand break repair genes The GO database was used to compile a list of 66 genes involved in DNA double-strand break repair. The human genome was searched for retrocopy copies of each of these. The number of repair genes with 0, 1, 2, 3, 4, or 5 retrocopy copies is shown. None of the 66 genes had more than 5 retrocopy copies.

We were interested in determining whether the presence of five retrocopies of *KU70* in the human genome is typical for a gene involved in double-stranded break repair. Because some gene families might be more or less prone to retrocopy formation and retention than others, we compared the number of retrocopies formed from *KU70* to other genes involved in DNA double-strand break repair. A list of all genes in the “double-strand break repair” biological process category (GO: 0006302) was compiled using the Gene Ontology (GO) database. Each was used as a query to identify retrocopy copies elsewhere in the human genome. A retrocopy was defined as any sequence match that (1) contains no introns, and (2) returns the parent gene when it itself is used to query the human genome (*i.e.*, the gene and retrocopy are reciprocal “best hits”). No criteria for conservation of the ORF was included, and some retrocopies appear to be degraded by mutation. In total, 51 double-strand break repair genes had no discernable retrocopies. Eleven genes [*MRE11*, *RAD21*, *FEN1*, *TRIP13*, *UBE2V2*, *PIR51 (RAD51AP1)*, *SHFM1 (SEM1)*, *BRCC3*, *RNF168*, *OBFC2B (NABP2)*, and *RTEL1*] had one retrocopy. Two genes (*SOD1* and *FAM175A*) had two retrocopies, and one gene (*UBE2N*) had four retrocopies. *KU70*, with five retrocopy copies, is the only one out of 66 with five retrocopy copies (Figure 3). Expression patterns of these genes in the ovary and testis indicate that the expression of *KU70* is the highest of all 66 genes in the testis and the second highest in the ovary (after *SOD1*) (Supplementary Figure S4). High expression of genes does not always lead to retrocopies as *CIB1*, *VCP*, and *KU80* all have high expression in the testis and ovary without the creation of retrocopies (Supplementary Figure S4).

### *NUKU2* has evolved under positive selection

There are three fates for any duplicated gene. A newly copied gene may be preserved by purifying selection if there is an adaptive advantage to having a second copy of the original gene. If the new gene copy is not expressed or confers no selective advantage, it will undergo neutral decay and accumulate point mutations and stop codons. Finally, if one of the duplicated genes is selected to evolve a novel function, this will occur through positive selection for advantageous mutations that arise and result in a period of relatively rapid sequence evolution in one of the copies. Each of these three fates can be read within the DNA sequence of duplicated genes after they have diverged. Looking at the evolutionary signatures recorded may offer clues as to the potential function of the retrocopy and how it may relate to the parent gene’s function. Specifically, patterns of accumulation of nonsynonymous versus synonymous mutational accumulation can be analyzed. Conserved genes like *KU70* would be expected to accumulate fewer nonsynonymous changes than synonymous changes (dN/dS < 1). If a retrocopy does not contribute to the fitness of the organism, it will accumulate these two types of changes at an equal rate (dN/dS = 1). However, if a retrocopy acquires a new function and is selected for optimization of this function, it would bear the signature of increased nonsynonymous mutation accumulation (dN/dS > 1).

**Figure 4:**
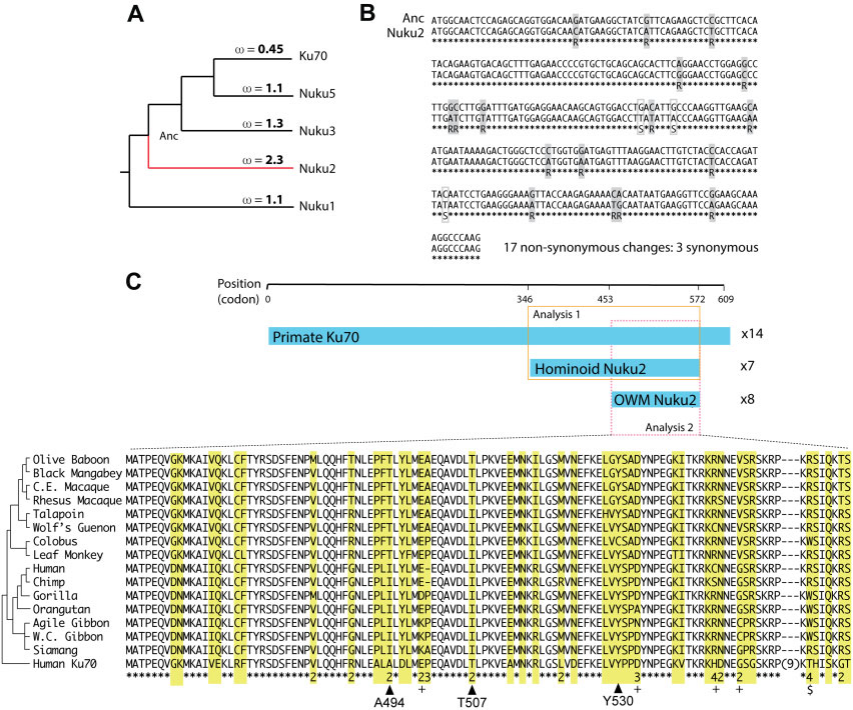
Molecular evolution of *KU70* retrocopies (A) Human *KU70* and four of the human *NUKU* retrocopies were aligned in the region of a common ORF. The branch-sites model assigned dN/dS values to each branch on the tree. These values summarize the evolution that has occurred since each retrocopy was formed. “Anc” refers to the node representing the formation of *NUKU2*, and the predicted sequence at this node was generated by codeml. (B) *NUKU2* is aligned to the “Anc” ancestral sequence in the region of the ORF which was analyzed in the analysis in panel A. Nonsynonymous changes and synonymous changes are illustrated by gray and white boxes, respectively, in the alignment. (C) *KU70* sequences were gathered for a total of 14 simian primate species, and *NUKU2* sequences were gathered from 15 species. All *NUKU2* sequences contain an ORF that is shorter than the *KU70* ORF, and it is even shorter in Old World monkeys than it is in hominoids. Two analyses of codon evolution were performed, one containing the sequences in the orange box (Analysis 1; longer ORF, *KU70* sequences plus 7 hominoid *NUKU2* sequence), and one containing the sequences in the pink box (Analysis 2; shorter ORF, *KU70* sequences plus all *NUKU2* sequences). The alignment shows the region that is an ORF in all genes. All *NUKU2* sequences are shown, with human *KU70* as an outgroup. In yellow are diverged sites, and numbers at the bottom indicate how many amino acid changes have occurred at those positions during *NUKU2* evolution (only indicated where dN/dS is greater than 1). The ‘$’ indicates a site that has changes from R to W three different times during *NUKU2* evolution. The ‘+’ sign indicates sites found to be under positive selection in the Analysis 1 branch-sites calculation (posterior probability > 0.5).

The increased number of *NUKU* retrocopies is likely due in part to high expression of *KU70*, but their retention could be rationalized if there is positive selection. The codeml program in the PAML package (Yang 2007) was used to analyze the selective pressures that have acted on each of the *NUKU* retrocopies since they were formed. A tree of the human *KU70* and *NUKU* retrocopies was analyzed by the branch-sites model (Figure 4A). The analysis of patterns of nonsynonymous and synonymous mutational accumulation can only be performed in ORFs, so a region at the C-terminal end of the retrocopies was analyzed because it is an ORF in all of the retrocopies except for *NUKU4*, which has experienced an *Alu* insertion in this region. The free-ratio model uses maximum likelihood to estimate a dN/dS ratio for each branch on the tree. As would be expected, the branch leading to *KU70* has a value of dN/dS = 0.45, indicating that nonsynonymous changes have accumulated at a rate less than half of the rate of synonymous changes (Figure 4A). Three of the pseudogenes, *NUKU1*, *NUKU3*, and *NUKU5*, have a dN/dS signature not statistically different from 1, indicating neutral evolution of these genes. However, the branch along which *NUKU2* has been evolving shows a dN/dS value of 2.3. We retrieved the predicted ancestral sequence from the node marked “Anc,” which is the prediction of the *NUKU2* sequence as it looked at the time of retrotransposition (Figure 4A). Comparing this to the extant *NUKU2* sequence (Figure 4B) allowed us to determine that 17 nonsynonymous mutations and three synonymous mutations have occurred in this region of the retrocopy since it was formed more than 30 MYA. We used Monte Carlo simulation to determine that this rate of evolution is significantly greater than the neutral expectation of dN/dS = 1 (*P* = 0.007) (Figure 4).

To further analyze the evolution of *NUKU2*, we determined the genetic sequence of *NUKU2* and *KU70* from 12 simian primate genomes (Supplementary Table S2). Because it is expressed in all tissue types and contains multiple introns, *KU70* was amplified and sequenced from mRNA, whereas *NUKU2* was amplified and sequenced from genomic DNA. These sequences were combined with those available from several primate species with sequenced genome projects (human, chimpanzee, orangutan, and rhesus macaque), and genes were also re-sequenced from these species where appropriate. Our analysis includes only Old World monkey and hominoid species as *NUKU2* has been largely deleted in the marmoset and squirrel monkey genomes (Supplementary Figure S2). Interestingly, the predicted ORF in human *NUKU2* (Figures 1A and 4C) was conserved in all hominoid species. In Old World monkeys, there was also a conserved *NUKU2* ORF, but it was shorter due to an upstream stop codon leading to the potential use of an alternative ATG codon further downstream (Figure 4C). Since *NUKU* ORFs were predicted to be under positive selection and not *KU70*, we used the branch-sites model and specified all of the *NUKU2* branches as the foreground clade (Zhang *et al.* 2005). This allowed us to look for positive selection of codons specifically in these species. Two analyses were performed, one with all *KU70* sequences and only the hominoid species where the longer reading frame was analyzed (orange box in Figure 4C), and one with all species where the shorter ORF was analyzed (pink box in Figure 4C). When the larger ORF was analyzed in hominoids only, it was estimated that 9% of the codons in *NUKU2* had a dN/dS of 7.05. Comparison to the null model shows the inference of positive selection to be statistically significant (*P* = 0.029; Table 1). Support is not as strong when the shorter ORF of *NUKU2* was analyzed across all species (*P* = 0.130), likely due to analyzing a shorter sequence of aligned DNAs.

**Table 1:** Molecular evolution of *NUKU2* in primates

Dataset^a^	Branch-site model	Estimate of parameters^b^		*Test 2*	
				2Δl^c^	*P*-value
Analysis 1 hominoid *NUKU2*	Model A with ω_2_ fixed at 1 Model A	l = −1,421.47 l = −1,419.10	p _0_ = 0.354 p_1_ = 0.375 p_2_ + p_3_ = 0.271 ω_0_ = 0.000 ω_1_ = 1.000 ω_2_ = 1.000 p _0_ = 0.455 p_1_ = 0.456 p_2_ + p_3_ = 0.089 ω_0_ = 0.000 ω_1_ = 1.000 ω_2_ = 7.054	4.75	*P* = 0.029
Analysis 2 OWM and hominoid *NUKU2*	Model A with ω_2_ fixed at 1 Model A	l = −853.17 l = −852.02	p _0_ = 0.353 p_1_ = 0.603 p_2_ + p_3_ = 0.044 ω_0_ = 0.000 ω_1_ = 1.000 ω_2_ = 1.000 p _0_ = 0.353 p_1_ = 0.516 p_2_ + p_3_ = 0.131 ω_0_ = 0.000 ω_1_ = 1.000 ω_2_ = 3.488	2.29	*P* = 0.130

a Both datasets included the *KU70* sequences from seven hominoids: *Homo sapiens*, *Gorilla gorilla*, *P. pygmaeus* (Sumatran orangutan), *P. pygmaeus* (Borneo orangutan), *Hylobates syndactylus*, *Hylobates leucogenys, H. agilis*, and from eight Old World monkeys: *Macaca mulatta*, *M. fascicularis*, *Lophocebus albigena*, *P. anubis*, *M. talapoin*, *C. wolfi*, *C. guereza*, *T. francoisi*. Both datasets also included *NUKU2* from the seven hominoids listed above as well as from chimpanzee (*Pan troglodytes*). Analysis 2 also included *NUKU2* from the eight Old World monkey species. In both analyses, the *NUKU2* clade was defined at the foreground clade and the *KU70* clade was defined at the background clade.

b Models were run using the f61 codon frequency model. l = ln of the likelihood.

c Twice the difference in the natural logs of the likelihoods (Δl × 2) of the two models being compared. This value is used in a likelihood ratio test along with the degrees of freedom (1 in this case). In Test 2, Model A, which allows positive selection on the foreground clade, is compared to a null model (Model A with ω_2_ fixed at 1). The *P*-value indicates the confidence with which the null model can be rejected.

### The tissue-specific expression and loss of *Ku80* interaction by *Nuku2*

**Figure 5:**
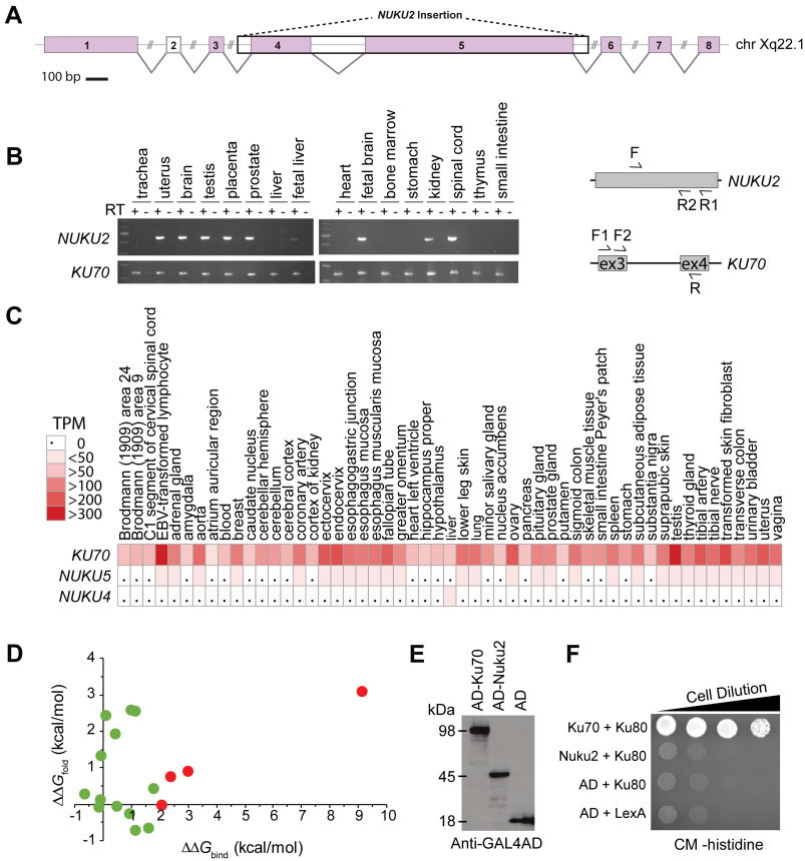
*NUKU* retrocopies are functionally distinct from *KU70*. (A) A lymphocyte-specific processed mRNA (EU224311) mapped to the human X chromosome with the insertion site of *NUKU2* boxed in black. Predicted splice sites are indicated between exons with 100% identity to the X chromosome (pink boxes). A significant match to exon 2 was not identified within the X chromosome. (B) RT-PCR was used to analyze the expression of *NUKU2* and *KU70* from total mRNA harvested from different human tissues. Nested primer pairs are shown to the right. The product of a first-round RT-PCR reaction (primers F—R1) was then amplified with a second set of primers (F and R2), where R2 sits interior to R1. All three primers were designed to be specific to transcripts from *NUKU2*, as the ultimate base at the 3’ end of the primer placed such that it pairs with a base that is unique to *NUKU2* relative to the other five retrocopies. *NUKU2* does not have introns, but the *KU70* primers span an intron. Nested PCR with specific primers was also used to amplify the *KU70* transcript, which is different in size from the product obtained from genomic DNA. (C) Relative tissue-specific expression patterns of *KU70*, *NUKU4*, and *NUKU5* measured in transcripts per million (TPM) (GTEx Consortium *et al.* 2017). (D) For each Nuku2p mutation within 5 Å of Ku80p, ΔΔ*G*_bind_ was plotted on the x-axis, whereas ΔΔ*G*_fold_ was plotted on the y-axis. Mutations are shown in green with *x*-axis values ΔΔ*G*_bind_ <2 kcal/mol and *y*-axis values -3 < ΔΔ*G*_fold_ < 3 kcal/mol are considered functional since they are likely to retain the ability to fold and bind. Mutations are shown in red with *x*-axis values ΔΔ*G*_bind_ >2 kcal/mol and *y*-axis values −3 < ΔΔ*G*_fold_ < 3 kcal/mol are predicted to retain folding but disrupt Ku80p binding. (E) Western blot confirming protein expression of each AD fusion construct in the yeast strains used for two-hybrid analysis. (F) A yeast two-hybrid test assaying the interaction of Ku70p or Nuku2p with Ku80p. The Gal4 AD is either fused to Ku70p (top row), Nuku2p (second row), or expressed alone (third and fourth rows). LexA is a DNA binding domain and is either fused to Ku80p (top three rows) or expressed alone (bottom row). A positive interaction enables growth on complete media (CM) lacking histidine.

Two human mRNA transcripts (EU224311 and ENST00000435236.2) were identified in the GenBank and Ensembl databases that verify the transcription and splicing of *NUKU2* on the X chromosome (Figure 5A). While we were unable to detect the spliced transcript of EU224311 by PCR, potentially because it is lymphocyte-specific, we performed 5’ and 3’ RACE and were successful in characterizing the structure of the unspliced transcript of *NUKU2* (ENST00000435236.2) from total RNA isolated from the human testis (Supplementary File S1). These transcripts are almost certainly derived from the transcription of the *NUKU2* locus due to 100% identity to the nucleotide sequence and putative translation products from *NUKU2* (Supplementary File S1 and Supplementary Table S1). To determine whether there were other *NUKU2* transcripts within other human tissues we designed PCR primers to specifically detect transcripts of the *NUKU2* retrocopy. We used nested PCR with *NUKU2*-specific primers, determined the genetic sequence of all products and confirmed that they were a perfect match only to the *NUKU2* retrocopy. As shown, *NUKU2* is expressed in uterus, brain, testis, placenta, prostate, fetal liver, fetal brain, kidney, and spinal cord (Figure 5B). We confirmed the absence of contaminating genomic DNA by performing RT-PCR reactions in which the RT had been omitted. We also amplified *KU70* by a similar nested strategy, using primers located in two neighboring exons, to distinguish by size products of RT-PCR from PCR products that may be produced from contaminating genomic DNA. No genomic DNA was detected by this assay. This ubiquitous tissue expression pattern of *KU70* reflects its function as an essential housekeeping gene and is shown in other published datasets (Figure 5C) (GTex project version 7) (GTEx Consortium *et al.* 2017). We also found evidence for the tissue-specific expression of both *NUKU4* and *NUKU5* and a transcript from *NUKU4* in the Ensembl database (ENST00000420392.1) (Figure 5C and Supplementary Table S1) (GTEx Consortium *et al.* 2017). These results confirm that *NUKU2*, *NUKU4*, and *NUKU5* are expressed in humans, expression is tissue-specific, and tissue-specificity has diverged from that of *KU70*, likely due to new regulatory signals at their new genomic location.

Ku70p is known to interact with Ku80p, thereby forming the Ku heterodimer that associates with broken ends of double-stranded DNA. To explore the potential biochemical function of a putative Nuku2 protein, compared to Ku70p, we examined the functional consequences of more than 10,000 mutational changes in Ku70p when bound to Ku80p using semi-empirical molecular modeling, as implemented by FoldX (Supplementary Figure S5 and Supplementary File S2) (Guerois *et al.* 2002; Schymkowitz *et al.* 2005). By comparing the amino acid changes between Ku70p and Nuku2p we individually modeled 27 nonsynonymous mutations that are present in *NUKU2* onto the heterodimeric co-crystal of Ku70p-Ku80p [PDB: 1JEY (Walker *et al.* 2001)] and measured the change in free energy for binding (ΔΔ*G*_bind_). The 11 mutations present in Nuku2p that were more than 5 Å from the Ku80p interface had an average ΔΔ*G*_bind_ of 0.04 kcal/mol (SD ± 0.17), indicating that these changes would not be expected to disrupt Ku80p binding (Supplementary Figure S5). The majority (81%) of the remaining 16 *NUKU2*-specific mutations that are within 5 Å of the Ku70p-Ku80p interface are also predicted to have little impact upon the interaction of these proteins (ΔΔ*G*_bind_ <2 kcal/mol; average 0.60 kcal/mol, SD ± 0.74) (Figure 5D; green data points). However, four mutations at this interface (G349V, F410L, A494I, and T507I) had a ΔΔ*G*_bind_ >2 kcal/mol (Figure 5D; red data points). This indicates that these mutations alone would be predicted to disrupt the binding of Ku70p to Ku80p, and therefore, in combination are likely to prevent binding of Nuku2p to Ku80p. In addition, because Nuku2p is predicted to be truncated relative to Ku70p, there would be a 39% reduction in the surface area available for Ku80p binding from ∼9500 to ∼5800 Å^2^, which would also reduce the likelihood of a Nuku2p-Ku80p interaction *(*PISA analysis, Krissinel 2015). Analysis of disruptive mutations in hominoid *NUKU2* shows the presence of the same G349V, A494I, and T507I mutations that are found in human *NUKU2*. Only T507I appears within the *NUKU2* gene of Old World monkeys, in addition to a single disruptive mutation unique to colobus monkey (Y530C; ΔΔ*G*_bind_ >2) (Figure 4). Finally, nonsynonymous mutations in *NUKU2* at sites under positive selection in primates have average ΔΔ*G*_bind_ and ΔΔ*G*_fold_ values of 0.33 (SD ± 0.45) and 1.00 (SD ± 1.46), respectively. This would suggest that these mutational changes were not driven by selection to disrupt Ku80p interaction or to alter Nuku2p folding.

Molecular modeling predicts that the truncation of *NUKU2* and several nonsynonymous mutations disrupt an interaction with Ku80p. To validate these *in silico* predictions we used the yeast two-hybrid *in vivo* protein interaction assay to test the interaction of either Ku70p or Nuku2p with Ku80p. Ku70p and Nuku2p were both fused to the Gal4 activation domain (AD), and each construct was co-transformed with a plasmid encoding the LexA-Ku80p fusion protein (Figure 5E). Co-transformants of AD-Ku70p and LexA-Ku80p were able to grow on media lacking histidine, signifying a positive interaction. AD-Nuku2p and LexA-Ku80p were unable to interact, and yeasts were unable to grow on histidine deficient plates. The LexA DNA binding-domain was also unable to interact with Ku80p or the AD (Figure 5F). Both the tissue-specific expression and inability to interact with Ku80p suggest that *NUKU2* has diverged from its parent gene *KU70* and potentially acquired new biological functions.

### Expression of *NUKU2* and *NUKU5* does not impact retrovirus transduction

Ku is known to be important for the replication of many different viruses, including mammalian retroviruses and retrotransposons (Daniel *et al.* 1999; Downs and Jackson 1999; Li *et al.* 2001; Suzuki *et al.* 2009; Zheng *et al.* 2011). We considered that Nuku proteins could act to antagonize viral replication by mimicking Ku70p and evidence of positive selection might suggest host-virus antagonism (Figure 4). To test whether the expression of *NUKUs* might disrupt the early stages of the retroviral lifecycle, we first confirmed the transient expression of *NUKU2* (human and rhesus macaque) and *NUKU5* (human) within the human HEK293T and HeLa cell lines (Supplementary Figure S6). Twenty-four hours post-transfection these cell lines were transduced with GFP using a single-cycle VSV-G pseudotyped human immunodeficiency virus 1 (HIV-1), feline immunodeficiency virus (FIV), and murine leukemia virus (MLV). Forty-eight hours post-infection the percentage GFP-expressing cells was measured using flow cytometry, and we found that *NUKU* expression did not affect retroviral transduction, relative to a control cell line expressing maltose-binding protein (Supplementary Figure S6).

### Evidence of *Nuku* gene translation and *Ku70-*like proteins

**Figure 6:**
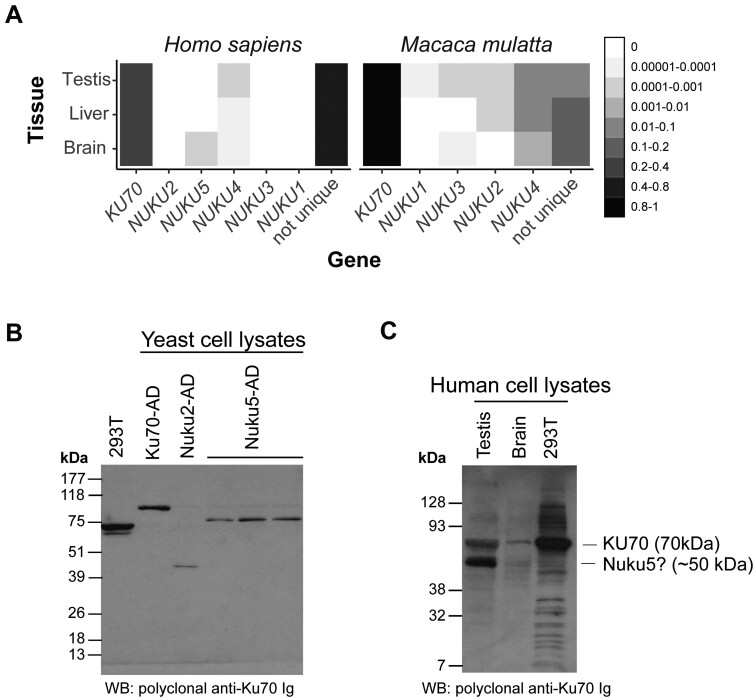
Detection of a putative *KU70* retrocopy-encoded protein (A) A heat map representation of Ribo-seq data reveals probable translation of several *NUKU* retrocopies. Many of the short (∼30bp) Ribo-seq reads identically match more than one gene and are classified as “not unique.” The remaining reads align uniquely to either *KU70* or one retrocopy. The mean proportion of reads aligning to each gene in three tissues is shown (*n* = 3). (B) Identification of an anti-Ku70 antibody that recognizes Nuku2p, Nuku5p, and Ku70p. The ORFs of *KU70*, *NUKU2*, and *NUKU5* were fused to the *GAL4* AD and expressed in yeast. A Western blot of these proteins shows that a single polyclonal anti-Ku70 antibody recognizes all three Nuku fusion proteins (three independent transformants of Nuku5-AD are shown). (C) Whole-cell protein lysates from testis, brain, and HEK293T cells were purchased or cultured. The anti-Ku70 antibody characterized in panel A was used to probe these extracts.

Since several *NUKUs* appear to be transcriptionally active, we wished to address if these retrocopies were capable of producing proteins. Ribosome profiling (Ribo-seq) data derived from large scale surveys of tissues from human and rhesus macaque were first assessed for unique *NUKU* transcripts that could be unambiguously identified by their sequence from *KU70* and other *NUKU* transcripts (Wang *et al.* 2020). There is evidence of transcripts from *NUKU4* and *NUKU5* associated with ribosomes in humans, and all *NUKU* transcripts in rhesus macaque (Figure 6A). In both species, *NUKU4* transcripts appeared to be more commonly associated with ribosomes in the three tissues surveyed.

We assume that the two retrocopies most likely to be expressed as proteins are Nuku2p and Nuku5p. The former has documented tissue-specific expression, a spliced transcript, and positive selection, and the latter, which is the youngest retrocopy, produces transcripts in many tissues, has the most intact ORF, and has evidence of translation in humans from Ribo-seq data. We screened several anti-Ku70 polyclonal antibodies for cross reactivity with Ku70p, Nuku2p, and Nuku5p. We used Gal4AD-Ku70 or Gal4AD-Nuku fusion proteins expressed in yeast to test this, and we identified an antibody that specifically recognized all three constructs (Figure 6B). The protein band in HEK293T cell extracts shows the position of untagged Ku70p, and this antibody does not appear to cross-react with the endogenous copy of Ku70p in yeast. The tagged copy of human Ku70-AD is larger than the untagged version (Figure 6B, lane 2 *vs* lane 1). The tagged versions of Nuku2p and Nuku5p are shorter, due to the truncated ORFs in these two genes (Figure 1A).

We detected high levels of *NUKU2* transcription in brain and testis among other tissues (Figure 5C). Therefore, we probed protein lysates from human brain tissue, testis tissue, and HEK293T cells with our anti-Ku70p antibody (Figure 6C). Protein lysates from HEK293T cells show only a single strong band at ∼70 kDa, the size of human Ku70p. This band is also evident in the testis and brain cell lysates. We did not detect a prominent band at ∼25 kDa in any of the samples, the predicted molecular weight of Nuku2p based on the transcript that we amplified by RACE (Supplementary File S1). However, cell lysates from brain and testis tissues, but not HEK293T cells, show a second band at the predicted size of human Nuku5p (∼50 kDa), with the band being more prominent in testis than in brain.

## Discussion

*KU70* is highly conserved across primates, which contrasts other genes that are required for DNA repair that have been found to be evolving rapidly within humans and yeasts, potentially in response to prior or current selective pressure from viruses and retrotransposons (Sawyer and Malik 2006; Demogines *et al.* 2010; Lou *et al.* 2014; Abdul *et al.* 2018). Despite the conservation of *KU70*, we describe the accumulation and diversification of *KU70*-derived retrocopies within humans and nonhuman primates (*NUKUs*). The contribution of retrocopies to *de novo* gene formation and the evolution of novel gene functions has been extensively documented in different organisms (Long and Langley 1993; Courseaux and Nahon 2001; Betrán *et al.* 2002; Wang *et al.* 2002). *KU70* appears to be unique regarding the number of retrocopies that it has birthed relative to other genes required for NHEJ in primates. In addition to the expansion of the *NUKUs* we have also detected the rapid evolution and functional divergence of these retrocopies during primate speciation. Evidence of transcription and the association of transcripts with ribosomes would suggest that these proteins are present within human and primate tissues.

NHEJ is an important mechanism for DNA double-strand break repair in cellular organisms and is also important for the replication of DNA viruses and retroviruses/retrotransposons that generate DNA intermediates during their lifecycles. There are examples of NHEJ DNA repair mechanisms helping or hindering viral replication (Weitzman *et al.* 2010). For example, lack of DNA-PK (DNA-dependent protein kinase holoenzyme, consisting of Ku70p, Ku80p, and DNA-PKcs) during HIV replication results in reduced viral integration and an increase in cellular apoptosis due to integrase-mediated DNA damage (Daniel *et al.* 1999; Li *et al.* 2001). Also, loss of Ku70p causes the proteasome-mediated degradation of the viral integrase (Zheng *et al.* 2011). Retrotransposons and adenovirus have also been shown to be sensitive to the loss of Ku (Downs and Jackson 1999; Suzuki *et al.* 2009; Frost *et al.* 2017). Bacteriophages encode Ku homologs that recruit other host DNA repair proteins and appear to protect phage DNA from degradation (Pitcher *et al.* 2006; Bhattacharyya *et al.* 2018). Furthermore, the hijack of NHEJ machinery is not specific to viruses as the bacterial pathogen *Rickettsia conorii* binds to cell surface-exposed Ku70p as its receptor for cell entry (Monferran *et al.* 2004; Martinez *et al.* 2005; Chan *et al.* 2009). In these cases, it is apparent that NHEJ machinery (including Ku70p) is aiding the replication and survival of viruses and bacteria. Conversely, there are many examples of DNA viruses that encode protein effectors that actively disrupt the function of NHEJ. Specifically, adenoviruses prevent the concatenation of their genomes by NHEJ machinery by producing the proteins E4-34 kDa and E4-11 kDa that bind DNA-PK and inhibit NHEJ (Boyer *et al.* 1999). Human T-cell leukemia virus type-1 proteins Tax and HBZ and the agnoprotein of JC virus bind and interfere with the function of DNA-PK, impairing DNA repair and aiding cellular transformation (Darbinyan *et al.* 2004; Durkin *et al.* 2008; Rushing *et al.* 2018). Viral proteins also block the activity of DNA-PK as a pattern recognition receptor that binds cytoplasmic DNAs triggering innate immune signaling mechanisms mediated by IFN regulatory factor 3 (IRF-3), TANK-binding kinase 1 (TBK1), and stimulator of interferon genes (STING) (Zhang *et al.* 2011; Ferguson *et al.* 2012; Sui *et al.* 2017). DNA-PK has been shown to be directly targeted by the vaccinia virus effectors C4 and C16 by binding Ku and preventing interaction with DNAs and triggering of innate immune signaling pathways (Peters *et al.* 2013; Scutts *et al.* 2018). The abundance of viruses and bacteria that subvert the function of DNA-PK suggests that the *NUKUs* could play a role as dominant-negative proteins that would bind viral effectors. It has already been shown in higher eukaryotes that a dominant negative Ku80p with an N-terminal extension [Ku80/Ku86-autoantigen-related protein-1 (KARP-1)] interferes with DNA-PKcs activity causing X-ray hypersensitivity when expressed in cell lines (Myung *et al.* 1997). However, molecular modeling studies and empirical binding assays show that Nuku2p does not bind Ku80p and would therefore not be predicted to assemble as a component of DNA-PK. Furthermore, *NUKU2* appears to have only maintained coding capacity within the C-terminal domain, which is required for binding to DNA, Mre11p, and Bax, whereas the N-terminal domain binds to DNA-PKcs and Ku80p (Figure 1; Goedecke *et al.* 1999; Subramanian *et al.* 2005). Therefore, we would expect that *NUKU2* would not influence DNA-PK function, V(D)J recombination, or telomere maintenance, but might still be competent as a transcription factor, or regulate apoptosis and NHEJ by binding Mre11p or Bax, respectively (Subramanian *et al*. 2005; Goedecke *et al*. 1999). The observed expansion and maintenance of *KU70* retrocopies and the rapid evolution of *NUKU2* could have been driven by evolutionary conflict with viruses or other pathogenic microorganisms free from the constrains of maintaining DNA repair of innate signaling functions. Indeed, the retrotransposition of genes involved in innate immunity can create new host restriction factors to fight rapidly evolving viruses (Sayah *et al.* 2004; Brennan *et al.* 2008; Wilson *et al.* 2008; Yang *et al.* 2020). Although we do not observe any significant effect of *NUKU* expression upon retrovirus transduction in tissue culture, it remains plausible that other viruses known to interfere with DNA-PK or directly interact with Ku70p (*i.e.*, JC virus agnoprotein or adenovirus E1A) might be sensitive to the presence of *NUKUs* (Darbinyan *et al.* 2004; Frost *et al.* 2017). Alternatively, as we have detected tissue-specific transcription from *NUKUs* it is also possible that they might have a function in the regulation of *KU70* expression as antisense transcripts (Tam *et al.* 2008). Altogether, these data suggest that primate-specific *NUKUs* are significantly altered compared to *KU70* in their expression and protein-coding capacity. Our analyses suggest that their structure and function differ from *KU70* and that they have evolved rapidly during primate speciation. However, it remains to be further investigated the biological function of these retrocopies, which is complicated by the multifaceted role of Ku70 in the cell.

## References

[jkab163-B1] Abdul F , Filleton F, Gerossier L, Paturel A, Hall J, et al 2018. Smc5/6 antagonism by hbx is an evolutionarily conserved function of hepatitis B virus infection in mammals. J Virol. 92:e00769–18. 29848586 10.1128/JVI.00769-18 PMC6069175

[jkab163-B2] Aravind L , Koonin EV. 2001. Prokaryotic homologs of the eukaryotic DNA-end-binding protein Ku, novel domains in the Ku protein and prediction of a prokaryotic double-strand break repair system. Genome Res. 11:1365–1374. 11483577 10.1101/gr.181001 PMC311082

[jkab163-B3] Benovoy D , Drouin G. 2006. Processed pseudogenes, processed genes, and spontaneous mutations in the Arabidopsis genome. J Mol Evol. 62:511–522. 16612535 10.1007/s00239-005-0045-z

[jkab163-B4] Betrán E , Wang W, Jin L, Long M. 2002. Evolution of the phosphoglycerate mutase processed gene in human and chimpanzee revealing the origin of a new primate gene. Mol Biol Evol. 19:654–663. 11961099 10.1093/oxfordjournals.molbev.a004124

[jkab163-B5] Bhattacharyya S , Soniat MM, Walker D, Jang S, Finkelstein IJ, et al 2018. Phage Mu Gam protein promotes NHEJ in concert with *Escherichia coli* ligase. Proc Natl Acad Sci USA. 115:E11614–E11622. 30487222 10.1073/pnas.1816606115 PMC6294893

[jkab163-B6] Boyer J , Rohleder K, Ketner G. 1999. Adenovirus E4 34k and E4 11k inhibit double strand break repair and are physically associated with the cellular DNA-dependent protein kinase. Virology. 263:307–312. 10544104 10.1006/viro.1999.9866

[jkab163-B7] Brennan G , Kozyrev Y, Hu S-L. 2008. TRIMCyp expression in Old World primates *Macaca nemestrina* and *Macaca fascicularis*. Proc Natl Acad Sci USA. 105:3569–3574. 18287033 10.1073/pnas.0709511105 PMC2265124

[jkab163-B8] Casola C , Betrán E. 2017. The genomic impact of gene retrocopies: what have we learned from comparative genomics, population genomics and transcriptomic analyses? Genome Biol Evol. 9:1351–1373. 28605529 10.1093/gbe/evx081 PMC5470649

[jkab163-B9] Chan YGY , Cardwell MM, Hermanas TM, Uchiyama T, Martinez JJ. 2009. Rickettsial outer-membrane protein B (rOmpB) mediates bacterial invasion through Ku70 in an actin, c-Cbl, clathrin and caveolin 2-dependent manner. Cell Microbiol. 11:629–644. 19134120 10.1111/j.1462-5822.2008.01279.x PMC2773465

[jkab163-B10] Comeron JM. 1999. K-Estimator: calculation of the number of nucleotide substitutions per site and the confidence intervals. Bioinformatics. 15:763–764. 10498777 10.1093/bioinformatics/15.9.763

[jkab163-B11] Courseaux A , Nahon JL. 2001. Birth of two chimeric genes in the Hominidae lineage. Science. 291:1293–1297. 11181993 10.1126/science.1057284

[jkab163-B12] Daniel R , Katz RA, Skalka AM. 1999. A role for DNA-PK in retroviral DNA integration. Science. 284:644–647. 10213687 10.1126/science.284.5414.644

[jkab163-B13] Darbinyan A , Siddiqui KM, Slonina D, Darbinian N, Amini S, et al 2004. Role of JC virus agnoprotein in DNA repair. J Virol. 78:8593–8600. 15280468 10.1128/JVI.78.16.8593-8600.2004 PMC479055

[jkab163-B14] Demogines A , East AM, Lee J-H, Grossman SR, Sabeti PC, et al 2010. Ancient and recent adaptive evolution of primate non-homologous end joining genes. PLoS Genetics. 6:e1001169. 20975951 10.1371/journal.pgen.1001169 PMC2958818

[jkab163-B15] Downs J , Jackson S. 1999. Involvement of DNA end-binding protein Ku in Ty element retrotransposition. Mol Cell Biol. 19:6260–6268. 10454572 10.1128/mcb.19.9.6260 PMC84583

[jkab163-B16] Durkin SS , Guo X, Fryrear KA, Mihaylova VT, Gupta SK, et al 2008. HTLV-1 Tax oncoprotein subverts the cellular DNA damage response via binding to DNA-dependent protein kinase. J Biol Chem. 283:36311–36320. 18957425 10.1074/jbc.M804931200 PMC2605996

[jkab163-B17] Esnault C , Maestre J, Heidmann T. 2000. Human LINE retrotransposons generate processed pseudogenes. Nat Genet. 24:363–367. 10742098 10.1038/74184

[jkab163-B18] Ferguson BJ , Mansur DS, Peters NE, Ren H, Smith GL. 2012. DNA-PK is a DNA sensor for IRF-3-dependent innate immunity. eLife. 1:1065–1017. 10.7554/eLife.00047 PMC3524801 23251783

[jkab163-B19] Frost JR , Olanubi O, Cheng SK-H, Soriano A, Crisostomo L, et al 2017. The interaction of adenovirus E1A with the mammalian protein Ku70/XRCC6. Virology. 500:11–21. 27769014 10.1016/j.virol.2016.10.004

[jkab163-B20] Gao YJ , Chaudhuri J, Zhu CM, Davidson L, Weaver DT, et al 1998. A targeted DNA-PKcs-null mutation reveals DNA-PK-independent functions for KU in V(D)J recombination. Immunity. 9:367–376. 9768756 10.1016/s1074-7613(00)80619-6

[jkab163-B21] Goedecke W , Eijpe M, Offenberg HH, van Aalderen M, Heyting C. 1999. Mre11 and Ku70 interact in somatic cells, but are differentially expressed in early meiosis. Nat Genet. 23:194–198. 10508516 10.1038/13821

[jkab163-B22] Gravel S , Larrivee M, Labrecque P, Wellinger RJ. 1998. Yeast Ku as a regulator of chromosomal DNA end structure. Science. 280:741–744. 9563951 10.1126/science.280.5364.741

[jkab163-B23] GTEx Consortium; Laboratory, Data Analysis &Coordinating Center (LDACC)—Analysis Working Group; Statistical Methods groups—Analysis Working Group; Enhancing GTEx (eGTEx) groups; NIH Common Fund; NIH/NCI; NIH/NHGRI; NIH/NIMH; NIH/NIDA; Biospecimen Collection Source Site—NDRI; Biospecimen Collection Source Site—RPCI; et al. 2017. Genetic effects on gene expression across human tissues. Nature. 550:204–213. 29022597 10.1038/nature24277 PMC5776756

[jkab163-B24] Gu YS , Jin SF, Gao YJ, Weaver DT, Alt FW. 1997. Ku70-deficient embryonic stem cells have increased ionizing radiosensitivity, defective DNA end-binding activity, and inability to support V(D)J recombination. Proc Natl Acad Sci USA. 94:8076–8081. 9223317 10.1073/pnas.94.15.8076 PMC21559

[jkab163-B25] Guerois R , Nielsen JE, Serrano L. 2002. Predicting changes in the stability of proteins and protein complexes: a study of more than 1000 mutations. J Mol Biol. 320:369–387. 12079393 10.1016/S0022-2836(02)00442-4

[jkab163-B26] Hall BG. 2013. Building phylogenetic trees from molecular data with MEGA. Mol Biol Evol. 30:1229–1235. 23486614 10.1093/molbev/mst012

[jkab163-B27] Harrison PM , Zheng D, Zhang Z, Carriero N, Gerstein M. 2005. Transcribed processed pseudogenes in the human genome: an intermediate form of expressed retrosequence lacking protein-coding ability. Nucleic Acids Res. 33:2374–2383. 15860774 10.1093/nar/gki531 PMC1087782

[jkab163-B28] Hsu HL , Gilley D, Blackburn EH, Chen DJ. 1999. Ku is associated with the telomere in mammals. Proc Natl Acad Sci USA. 96:12454–12458. 10535943 10.1073/pnas.96.22.12454 PMC22947

[jkab163-B29] Jin SF , Weaver DT. 1997. Double-strand break repair by Ku70 requires heterodimerization with Ku80 and DNA binding functions. EMBO J. 16:6874–6885. 9362500 10.1093/emboj/16.22.6874 PMC1170290

[jkab163-B30] Kaessmann H , Vinckenbosch N, Long M. 2009. RNA-based gene duplication: mechanistic and evolutionary insights. Nat Rev Genet. 10:19–31. 19030023 10.1038/nrg2487 PMC3690669

[jkab163-B31] Krissinel E. 2015. Stock-based detection of protein oligomeric states in jsPISA. Nucleic Acids Res. 43:W314–W319. 25908787 10.1093/nar/gkv314 PMC4489313

[jkab163-B32] Li L , Olvera JM, Yoder KE, Mitchell RS, Butler SL, et al 2001. Role of the non-homologous DNA end joining pathway in the early steps of retroviral infection. EMBO J. 20:3272–3281. 11406603 10.1093/emboj/20.12.3272 PMC150207

[jkab163-B33] Long M , Betrán E, Thornton K, Wang W. 2003. The origin of new genes: glimpses from the young and old. Nat Rev Genet. 4:865–875. 14634634 10.1038/nrg1204

[jkab163-B34] Long M , Langley CH. 1993. Natural selection and the origin of jingwei, a chimeric processed functional gene in Drosophila. Science. 260:91–95. 7682012 10.1126/science.7682012

[jkab163-B35] Lou DI , McBee RM, Le UQ, Stone AC, Wilkerson GK, et al 2014. Rapid evolution of BRCA1 and BRCA2 in humans and other primates. BMC Evol Biol. 14:155. 25011685 10.1186/1471-2148-14-155 PMC4106182

[jkab163-B36] Maestre J , Tchénio T, Dhellin O, Heidmann T. 1995. mRNA retroposition in human cells: processed pseudogene formation. EMBO J. 14:6333–6338. 8557053 10.1002/j.1460-2075.1995.tb00324.x PMC394758

[jkab163-B37] Marques AC , Dupanloup I, Vinckenbosch N, Reymond A, Kaessmann H. 2005. Emergence of young human genes after a burst of retroposition in primates. PLoS Biol. 3:e357. 16201836 10.1371/journal.pbio.0030357 PMC1251493

[jkab163-B38] Martinez JJ , Seveau S, Veiga E, Matsuyama S, Cossart P. 2005. Ku70, a component of DNA-dependent protein kinase, is a mammalian receptor for *Rickettsia conorii*. Cell. 123:1013–1023. 16360032 10.1016/j.cell.2005.08.046

[jkab163-B39] Milne GT , Jin SF, Shannon KB, Weaver DT. 1996. Mutations in two Ku homologs define a DNA end-joining repair pathway in *Saccharomyces cerevisiae*. Mol Cell Biol. 16:4189–4198. 8754818 10.1128/mcb.16.8.4189 PMC231416

[jkab163-B40] Monferran S , Paupert J, Dauvillier S, Salles B, Muller C. 2004. The membrane form of the DNA repair protein Ku interacts at the cell surface with metalloproteinase 9. EMBO J. 23:3758–3768. 15385961 10.1038/sj.emboj.7600403 PMC522801

[jkab163-B41] Myung K , He DM, Lee SE, Hendrickson EA. 1997. KARP-1: a novel leucine zipper protein expressed from the Ku86 autoantigen locus is implicated in the control of DNA-dependent protein kinase activity. EMBO J. 16:3172–3184. 9214634 10.1093/emboj/16.11.3172 PMC1169935

[jkab163-B42] Nussenzweig A , Chen CH, Soares VD, Sanchez M, Sokol K, et al 1996. Requirement for Ku80 in growth and immunoglobulin V(D)J recombination. Nature. 382:551–555. 8700231 10.1038/382551a0

[jkab163-B43] Ohshima K , Hattori M, Yada T, Gojobori T, Sakaki Y, et al 2003. Whole-genome screening indicates a possible burst of formation of processed pseudogenes and Alu repeats by particular L1 subfamilies in ancestral primates. Genome Biol. 4:R74. 14611660 10.1186/gb-2003-4-11-r74 PMC329124

[jkab163-B44] Peters NE , Ferguson BJ, Mazzon M, Fahy AS, Krysztofinska E, et al 2013. A mechanism for the inhibition of DNA-PK-mediated DNA sensing by a virus. PLoS Pathogens. 9:e1003649. 24098118 10.1371/journal.ppat.1003649 PMC3789764

[jkab163-B45] Pitcher RS , Tonkin LM, Daley JM, Palmbos PL, Green AJ, et al 2006. Mycobacteriophage exploit NHEJ to facilitate genome circularization. Mol Cell. 23:743–748. 16949369 10.1016/j.molcel.2006.07.009

[jkab163-B46] Rosikiewicz W , Kabza M, Kosiński JG, Ciomborowska-Basheer J, Kubiak MR, et al 2017. RetrogeneDB–a database of plant and animal retrocopies. Database. 2017: bax038. 10.1093/database/bax038 PMC5509963 29220443

[jkab163-B47] Roth DB , Porter TN, Wilson JH. 1985. Mechanisms of nonhomologous recombination in mammalian cells. Mol Cell Bio. 5:2599–2607. 3016509 10.1128/mcb.5.10.2599-2607.1985 PMC366995

[jkab163-B48] Rushing AW , Hoang K, Polakowski N, Lemasson I. 2018. The human T-cell leukemia virus type 1 basic leucine zipper factor attenuates repair of double-stranded DNA breaks via nonhomologous end joining. J Virol. 92:e00672–18. 29769340 10.1128/JVI.00672-18 PMC6052317

[jkab163-B49] Sawyer SL , Malik HS. 2006. Positive selection of yeast nonhomologous end-joining genes and a retrotransposon conflict hypothesis. Proc Natl Acad Sci USA. 103:17614–17619. 17101967 10.1073/pnas.0605468103 PMC1693795

[jkab163-B50] Sayah DM , Sokolskaja E, Berthoux L, Luban J. 2004. Cyclophilin A retrotransposition into TRIM5 explains owl monkey resistance to HIV-1. Nature. 430:569–573. 15243629 10.1038/nature02777

[jkab163-B51] Schacherer J , Tourrette Y, Souciet JL, Potier S, de Montigny J. 2004. Recovery of a function involving gene duplication by retroposition in *Saccharomyces cerevisiae*. Genome Res. 14:1291–1297. 15231745 10.1101/gr.2363004 PMC442144

[jkab163-B52] Schlötterer C. 2015. Genes from scratch – the evolutionary fate of *de novo* genes. Trends Genet. 31:215–219. 25773713 10.1016/j.tig.2015.02.007 PMC4383367

[jkab163-B53] Schymkowitz J , Borg J, Stricher F, Nys R, Rousseau F, et al 2005. The FoldX web server: an online force field. Nucleic Acids Res. 33:W382–W388. 15980494 10.1093/nar/gki387 PMC1160148

[jkab163-B54] Scutts SR , Ember SW, Ren H, Ye C, Lovejoy CA, et al 2018. DNA-PK is targeted by multiple vaccinia virus proteins to inhibit DNA sensing. Cell Reports. 25:1953–1965.e4. 30428360 10.1016/j.celrep.2018.10.034 PMC6250978

[jkab163-B55] Sisu C , Pei B, Leng J, Frankish A, Zhang Y, et al 2014. Comparative analysis of pseudogenes across three phyla. Proc Natl Acad Sci USA. 111:13361–13366. 25157146 10.1073/pnas.1407293111 PMC4169933

[jkab163-B56] Stabell AC , Hawkins J, Li M, Gao X, David M, et al 2016. Non-human primate schlafen11 inhibits production of both host and viral proteins. PLoS Pathog. 12:e1006066. 28027315 10.1371/journal.ppat.1006066 PMC5189954

[jkab163-B57] Subramanian C , Opipari AW, Bian X, Castle VP, Kwok RPS. 2005. Ku70 acetylation mediates neuroblastoma cell death induced by histone deacetylase inhibitors. Proc Natl Acad Sci USA. 102:4842–4847. 15778293 10.1073/pnas.0408351102 PMC555706

[jkab163-B58] Sui H , Zhou M, Imamichi H, Jiao X, Sherman BT, et al 2017. STING is an essential mediator of the Ku70-mediated production of IFN-gimel 1 in response to exogenous DNA. Sci Signal. 10:eaah5054. 28720717 10.1126/scisignal.aah5054

[jkab163-B59] Suzuki J , Yamaguchi K, Kajikawa M, Ichiyanagi K, Adachi N, et al 2009. Genetic evidence that the non-homologous end-joining repair pathway is involved in LINE retrotransposition. PLoS Genet. 5:e1000461. 19390601 10.1371/journal.pgen.1000461 PMC2666801

[jkab163-B60] Tam OH , Aravin AA, Stein P, Girard A, Murchison EP, et al 2008. Pseudogene-derived small interfering RNAs regulate gene expression in mouse oocytes. Nature. 453:534–538. 18404147 10.1038/nature06904 PMC2981145

[jkab163-B61] Walker JR , Corpina RA, Goldberg J. 2001. Structure of the Ku heterodimer bound to DNA and its implications for double-strand break repair. Nature. 412:607–614. 11493912 10.1038/35088000

[jkab163-B62] Wang W , Brunet FG, Nevo E, Long M. 2002. Origin of sphinx, a young chimeric RNA gene in *Drosophila melanogaster*. Proc Natl Acad Sci USA. 99:4448–4453. 11904380 10.1073/pnas.072066399 PMC123668

[jkab163-B63] Wang Z-Y , Leushkin E, Liechti A, Ovchinnikova S, Mößinger K, et al 2020. Transcriptome and translatome co-evolution in mammals. Nature. 588:642–647. 33177713 10.1038/s41586-020-2899-z PMC7116861

[jkab163-B64] Weitzman MD , Lilley CE, Chaurushiya MS. 2010. Genomes in conflict: maintaining genome integrity during virus infection. Annu Rev Microbiol. 64:61–81. 20690823 10.1146/annurev.micro.112408.134016

[jkab163-B65] Weller GR , Kysela B, Roy R, Tonkin LM, Scanlan E, et al 2002. Identification of a DNA nonhomologous end-joining complex in bacteria. Science. 297:1686–1689. 12215643 10.1126/science.1074584

[jkab163-B66] Wilson SJ , Webb BLJ, Ylinen LMJ, Verschoor E, Heeney JL, et al 2008. Independent evolution of an antiviral TRIMCyp in rhesus macaques. Proc Natl Acad Sci USA. 105:3557–3562. 18287035 10.1073/pnas.0709003105 PMC2265179

[jkab163-B67] Yang Z. 2007. PAML 4: phylogenetic analysis by maximum likelihood. Mol Biol Evol. 24:1586–1591. 17483113 10.1093/molbev/msm088

[jkab163-B68] Yang L , Emerman M, Malik HS, McLaughlin RN. 2020. Retrocopying expands the functional repertoire of APOBEC3 antiviral proteins in primates. eLife. 9:e58436. 32479260 10.7554/eLife.58436 PMC7263822

[jkab163-B69] Zhang X , Brann TW, Zhou M, Yang J, Oguariri RM, et al 2011. Cutting edge: Ku70 is a novel cytosolic DNA sensor that induces type III rather than type I IFN. J Immunol. 186:4541–4545. 21398614 10.4049/jimmunol.1003389 PMC3720676

[jkab163-B70] Zhang ZL , Harrison PM, Liu Y, Gerstein M. 2003. Millions of years of evolution preserved: a comprehensive catalog of the processed pseudogenes in the human genome. Genome Res. 13:2541–2558. 14656962 10.1101/gr.1429003 PMC403796

[jkab163-B71] Zhang J , Nielsen R, Yang Z. 2005. Evaluation of an improved branch-site likelihood method for detecting positive selection at the molecular level. Mol Biol Evol. 22:2472–2479. 16107592 10.1093/molbev/msi237

[jkab163-B72] Zheng Y , Ao Z, Wang B, Jayappa KD, Yao X. 2011. Host protein Ku70 binds and protects HIV-1 integrase from proteasomal degradation and is required for HIV replication. J Biol Chem. 286:17722–17735. 21454661 10.1074/jbc.M110.184739 PMC3093848

